# Hospital News

**Published:** 1922-05

**Authors:** 


### HOSPITAL NEWS.

Lady de Frece (Miss Vesta Tilley) has been elected president of the Birmingham Childrens' Hospital.

The War Memorial of Burradon, a Northumbrian mining village, is a four-roomed cottage for the district nurse. The cottage has been built for the purpose.

The Raleigh Cycle Company has presented a specially designed bicycle for the use of the maternity department at St. Bartholomew's Hospital. The machine is fitted with front and rear carriers for the doctor's outfit.

The Leeds General Infirmary, which reports a deficiency of over £25,000, is looking forward to a visit from the Prince of Wales in the early summer to open the King Edward VII. Memorial Extension.

The Post Office has given £8 7s. 6d. to King's College Hospital in exchange for the old, defaced and damaged coins, which could not be passed through the bank, found in collecting boxes placed on the railings outside the hospital.

The Wolverhampton General Hospital has decided to appoint a sister-tutor and to form a preliminary training school for nurses in an annexe to the nurses' house. This decision will increase the nursing staff and shorten its hours.

The late Mr. J. P. Cumine was for fifty years secretary of the Aberdeen Dispensary and Vaccine Institution. Since 1840 the secretarial duties have been performed by partners in the same firm, of which the new secretary, Mr. Sands, is also a member.

Miss Murray has resigned the presidency of the Richmond District Nursing Association, which she has held for many years. She was one of the principal workers who in 1884 first formed the society, and has consented still to act as one of its vice-presidents.

The Ernest Edward Heywood Memorial Nurses' Home Extension at the Huddersfield Royal Infirmary has been opened by one of the donors, Mrs. Heywood. It provides rooms for twenty more nurses. In 1910 Mrs. Heywood opened the original home, which she and her husband had given in memory of their son.

The allegations of understaffing at Norwich Workhouse Infirmary, which were supported by Dr. Barclay, the medical officer, have led the house committee to recommend his suspension; and he has now resigned. Critics of the infirmary have suggested an inquiry by the Ministry of Health into the adequacy of the nursing staff.

Princess Marie Louise visited 30a Wimpole Street, on March 22, and there presented Miss Hellis, late Secretary of the London Throat Hospital, Great Portland Street, with a purse of 150 guineas, subscribed by the medical staff, members of committee, and friends of her old hospital, on her retirement, as a token of appreciation of her loyal and devoted services for 25 years.

Lady Thomas, wife of Sir W. J. Thomas, of Cardiff, has endowed a cot at the North Staffordshire Infirmary, where she was trained. As Miss Mary Maud Cooper, Lady Thomas was sister of Ward 6 and of the children's ward before going to Cardiff, where she was assistant-matron at King Edward VII. Hospital at the time of her marriage in 1917. Sir William Thomas, it will be recalled, crowned his benefactions to voluntary hospitals with the gift of £100,000 for the buildings of the Welsh Medical School.

The late Miss Amy Hill was for over forty-four years an active worker for the Royal Northern Hospital, Holloway. She became associated with the institution when, as the Great Northern Hospital, it occupied a small house in Caledonian Road, N., on the site of the Public Baths. The hospital apparently was the first in London to form a ladies' association and to admit ladies to the Committee of Management. Miss Hill became a member of the former in 1882 and of the latter in 1906. She saw the hospital's activities transferred to Holloway Road (with 64 beds) in 1888, the erection of the Reckitt Convalescent Home, Clacton-on-Sea, in 1909, the opening of the Hospital of Recovery, Southgate, last year, and the amalgamation of the Royal Chest Hospital, City Road—the oldest chest hospital—with the main institution. One of her chief interests was the new nurses' home.

Beckenham Cottage Hospital is to be enlarged by a large ward and eight smaller ones for paying patients. The kitchens will also be remodelled.

A cheque for £221 has been presented to Nurse Law in recognition of her services to the Berwick and District Nursing Association since its foundation 30 years ago.

An arm-chair and a sewing machine have been presented to Sister Fairchild by the committee of the Bristol and Clifton District Nurses' Society in recognition of her twenty-five years' devoted service.

The erection of a children's bungalow ward has been approved at the Royal Devon and Exeter Hospital; an army hut has been bought to supply some of the materials. A sum of £3,300 is at the committee's disposal for this purpose.

The dispute between the Hammersmith Board of Guardians and the Ministry of Pensions over the rental of the workhouse and infirmary buildings at Ducane Road as a special surgical hospital for disabled soldiers has been settled. A rent of £12,000 a year for two years from January 1, 1922, is to be paid.

A fancy fair in aid of the British Home and Hospital for Incurables, Crown Lane, Streatham, is to be held in the grounds of the institution on Wednesday, June 7. Particulars can be obtained from the organiser, Mrs. Frank Cripps, 1 Norfolk Street, Park Lane, W.1.

Christ Church Vicarage, Bradford, which for years has been the nurses' home at the Eye and Ear Hospital, must now be bought or surrendered. If the £2,000 now appealed for is not raised, the hospital will be dispossessed of a building the site of which is conveniently placed at its rear.

Bristol General Hospital has started a vaccine therapy department, and made arrangements for an out-patient mental clinic, where those suffering from incipient mental disease can be treated without incurring certification. The clinic is under the charge of Dr. J. V. Blachford, Medical Superintendent of the Bristol Mental Hospital.

Speaking at the annual meeting of the Edinburgh Hospital and Dispensary for Women, Mrs. J. T. Hunter said that there was a distinct reaction in Scotland against women doctors. In Glasgow, as far as they could see, there was not the least prospect for a young woman doctor to become a teacher on a hospital staff. The only solution was for women to have hospitals of their own.

Miss Van Thol has been appointed teacher of elocution to patients being treated for cleft palate at the Royal Dental Hospital, Leicester Square. The report states that original difficulties in speaking generally continue after treatment as an acquired habit, and that the help of a skilled elocutionist makes all the difference to patients when seeking employment.

Mr. Robert Edger, secretary of the Hartlepools Hospital has resigned his post, which he has held for the long period of twenty-six years. Mr. Edger's resignation was accepted with regret, and he was described as "a good and faithful servant." Since the hospital was established, in 1865, he has seen many changes which culminated in the additions and improvements made in 1920.

The first woman resident medical officer has been appointed to the staff of Ancoats Hospital, Manchester, in the person of Dr. Phyllis M. Congday, a graduate of Manchester University. The only women in resident posts hitherto have been on the staffs of special hospitals in Manchester. In the general hospitals they have been non-resident officers assisting in the out-patient departments.

After thirty years' work Horsham Hospital has dropped the style of cottage hospital, and is to be rebuilt on a new site adjoining the existing building. The plans have been prepared by Mr. F. G. Troup, F.R.I.B.A., and provide for two wards with twelve beds apiece, three private wards, and complementary staff quarters and offices. The cost is expected to be £13,000, of which £12,000 is in hand, apart from the value of the old building. The new scheme will rather more than double the hospital's accommodation, and the money for it leaves the endowment fund untouched.

The Infant Hospital, Dundee, situated at Broughty Ferry is the only one of its kind in Scotland, for it is not a hospital for sick children, but for the treatment of feeble-minded infants. Mr. R. Still declared at the recent annual meeting that the parents of the children under treatment learnt much, and that the hospital provided a field for medical research. Last year 160 patients were treated, many of whom are said to have suffered from the effects of unsuitable diet. Apart from the debt of £1,100 on the capital account, the revenue almost balances the expenses, but the committee feels that the institution should be more widely known both to the practitioners and the people of Dundee.

The Leeds Board of Guardians has arranged with the General Infirmary for the services of consultants, and also for medical students and post-graduates to attend the Union Infirmary by arrangement with the Medical Superintendent. The ordinary work of the Union Infirmary is not to be interrupted, and the Guardians reserve the right to terminate the agreement at any time. A somewhat similar arrangement in London is that by which the Medical School Committee of S. Mary's Hospital has received permission from the Paddington Guardians for the medical students to visit the wards of the Infirmary and for members of the staff of St. Mary's Hospital to give clinical teaching in the wards.

The committee of St. Peter's Hospital for urinary diseases has arranged with the Hampstead Guardians for 20 patients to be received at the New End Infirmary. St. Peter's Hospital had to close for repairs, and there was a long list of patients waiting admission. 20 patients, therefore, are at New End at a cost to the Hospital of £3 ahead a week. The Hospital provides its own medical staff and nurses. The arrangement is understood to benefit the rates, since the cost to the Guardians is £2 a week. The patients are accommodated in a ward which would otherwise be empty. Every example of mutually satisfactory co-operation is welcome, and encourages further accommodation of the kind.

The growing use of the Birmingham Poor Law Hospitals has led the Birmingham Guardians to form a settled policy. They invite the voluntary hospitals to restrict transfers to the Guardians' Infirmaries to persons resident in the Birmingham Union, or to persons residing in those other Unions which have agreed to pay for the maintenance of cases coming from their areas. Not all the local Guardians have so agreed; and the Birmingham Guardians disclaim responsibility for patients from these areas unable to find admission to the voluntary hospitals. These rules, we understand, are not intended to apply to accidents occurring in the City. The high rate of admissions to the Dudley Road and Selby Oak Hospitals show that there is now apparently no prejudice against these Institutions.

On March 23 the Queen opened the new hospital buildings of the British Hospital for Mothers and Babies, Woolwich, and patients arrived a week later. It will be recalled that the hospital was the result of an amalgamation in 1915 between the British Lying-in Hospital and the Home for Mothers and Babies. Its aim is to serve as a National Training School for District Midwives. During the past year £20,000 was received and spent from the Ministry of Health, and though the buildings are far from complete, the ward block, a corner of the administration block and the chapel have been finished. The present Wood Street quarters have to be kept as a Nurses' Home. The hospital's scheme for relieving the unemployed by setting them to dig the rest of the basement and to make the front road, despite the Borough Council's support, fell through. It indicates, however, the eagerness with which the completion of its building scheme is desired.

---

Messrs. Manlove, Alliott & Co., Ltd., engineers, of Nottingham, have issued a catalogue (No. 650A) of their latest high-pressure steam disinfectors of various types for hospitals and institutions. Another catalogue (No. 512) shows a full range of their hydro-extractors or centrifugals for laundry work. Copies of these catalogues, which are very well produced, may be obtained on application to the company.

### Other News.

After 26 years' service, Dr. Francis Stevens, Medical Officer of Health for Camberwell, has retired.

The Central Association for the Care of the Mentally Defective, 24 Buckingham Palace Road, S.W.1, will be known in future as the Central Association for Mental Welfare.

We regret to hear of the death of Miss Elizabeth Mary Jones, H.M. Inspector (Ministry of Health) for Wales and border counties, formerly for many years Lady Superintendent and Matron of the Liverpool Royal Infirmary.

The Council of the College of Nursing has passed a resolution recording its appreciation of the action of the Minister of Health in approving the rules of the General Nursing Council for England and Wales, as amended on February 17. It feels confident that the rules as now amended are in the best interests of trained nurses.

During 1921 no fewer than 48,000 patients were treated at the several infectious hospitals of the Metropolitan Asylums Board. Some of the patients were admitted on more than one occasion. The number of patients was 5,423 on September 3, and exceeded the maximum number of cases under treatment on any one day in nineteen out of the last twenty-five years. The number rose rapidly until November 8, when the maximum, 9,599, was reached.

Sir Humphry Davy Rolleston has been appointed President of the Royal College of Physicians, in succession to Sir Norman Moore, who has resigned. He is Emeritus Physician to St. George's Hospital, Physician to the Victoria Hospital for Children, Chelsea, Consulting Physician to West Herts Infirmary, member of the Medical Administrative Committee, Royal Air Force; Visitor to King Edward VII. Memorial Fund, and Examiner in the Universities of Oxford, Cambridge, London, Durham, Manchester and Sheffield.

A Fellowship for the encouragement of research in Preventive Medicine has been instituted in memory of the late Auguste Sheridan Delépine, Professor of Public Health and Bacteriology in the University of Manchester from 1891 to 1921, by the addition of the emoluments of the former Junior Research Fellowships in Public Health to the interest derived from an endowment of £1,000 made by Dr. Charles Slater, of Tunbridge Wells. A Fellowship of £300 will be offered biennially and be open to competition by graduates in medicine of this or any other approved University, or who hold an approved registrable medical qualification.

The Joint Council of the Order of St. John and the British Red Cross Society, in conjunction with the Federation of Women's Institutes, has arranged a series of seven lectures on "How to Keep Well." The lectures are being given free each week to Women's Institutes in the counties of Surrey, Hampshire, Norfolk, Huntingdon, Stafford, East Sussex, Dorset, Essex and Lincolnshire, by specially-qualified women, to each of whom is allotted a circuit of ten institutes. All the expenses of the courses are borne by the Joint Council, with the exception of travelling expenses between the institutes and hospitality for which the Federation of Women's Institutes is responsible.

We have received a copy of the provisional programme of the Congress of Radiology and Physiotherapy, to be held in London on June 7–10. This Congress, organised by the Section of Electro-Therapeutics of the Royal Society of Medicine and the British Association for the Advancement of Radiology and Physiotherapy, is the postponed Bilingual Congress of 1921, intended as a compliment to French and Belgian confrères who entertained British representatives a small gathering at the end of the war. The subjects to be discussed include "Deep Therapy by X-Rays and Radium"; "The Action of the Direct Current on the Tissues in Health and Disease"; "The Uses of Electrical Methods in the Diagnosis and Prognosis of Paralysis"; "The Physiological Action and Therapeutic Uses of High Frequency Currents"; "The Re-education of Muscles"; "Cardiac Disorders"; and "Sciolosis." There will be an exhibition of X-Ray and electrical apparatus. Applications for membership should be sent to the hon. treasurer, Dr. James Metcalfe, 123 Harley Street, W1. The subscription for British members is £2 2s.

